# A case of one‐stage laparoscopic drainage and ureterocystoneostomy for iatrogenic ureteral injury associated with infected urinoma

**DOI:** 10.1002/iju5.12635

**Published:** 2023-09-03

**Authors:** Toshifumi Takahashi, Kouhei Maruno, Tatsuya Hazama, Hideto Ota, Yuya Yamada, Masakazu Nakashima, Kazuro Kikkawa, Masahiro Tamaki, Noriyuki Ito

**Affiliations:** ^1^ Department of Urology Japanese Red Cross Wakayama Medical Center Wakayama Japan

**Keywords:** iatrogenic ureteral injury, infected urinoma, laparoscopic drainage, laparoscopic ureterocystoneostomy, one‐stage surgery

## Abstract

**Introduction:**

Iatrogenic ureteral injury is a rare but often encountered complication of abdominal and gynecological surgery. This is a case of iatrogenic ureteral injury with infected urinoma treated with one‐stage laparoscopic drainage and ureterocystoneostomy.

**Case presentation:**

An 80‐year‐old man with rectal cancer had robot‐assisted low anterior rectum resection and left lateral lymph node dissection after colostomy and preoperative chemoradiotherapy. On the 14th postoperative day, he had a fever, and a noncontrast‐enhanced computed tomography scan revealed a low‐density polycystic area in the left pelvic cavity. Retrograde pyelography revealed contrast medium leaking from the left lower ureter, preventing ureteral stent placement. We identified it as a delayed ureteral injury with infected urinoma and performed laparoscopic one‐stage drainage and ureterocystoneostomy.

**Conclusion:**

This study reported a case of one‐stage laparoscopic drainage and ureterocystoneostomy for iatrogenic ureteral injury with infected urinoma.

**Keynote message**

One‐stage drainage and ureteral ureterocystoneostomy may prevent the decline in QOL associated with nephrostomy tube management, improve overall condition, and provide early therapeutic intervention for iatrogenic ureteral injury associated with infected urinoma.

**Abbreviations & Acronyms**
CRP: C‐reactive protein
EAU: European Association of Urology
NCCT: noncontrast enhanced computer tomography
QOL: quality of life
RP: retrograde pyelography
WBC: white blood cell

## Introduction

Iatrogenic ureteral injury is a rare but often encountered complication of abdominal and gynecological surgery.^1^, ^2^ Iatrogenic ureteral injury can result in ureteral stricture, urinoma, or urinary tract infection. It may require ureteral stent, nephrostomy tube management, urinoma drainage, and, if necessary, urinary tract reconstruction.

Here, we report a case of one‐stage laparoscopic drainage and ureterocystoneostomy for iatrogenic ureteral injury with infected urinoma.

## Case presentation

**Fig. 1:**
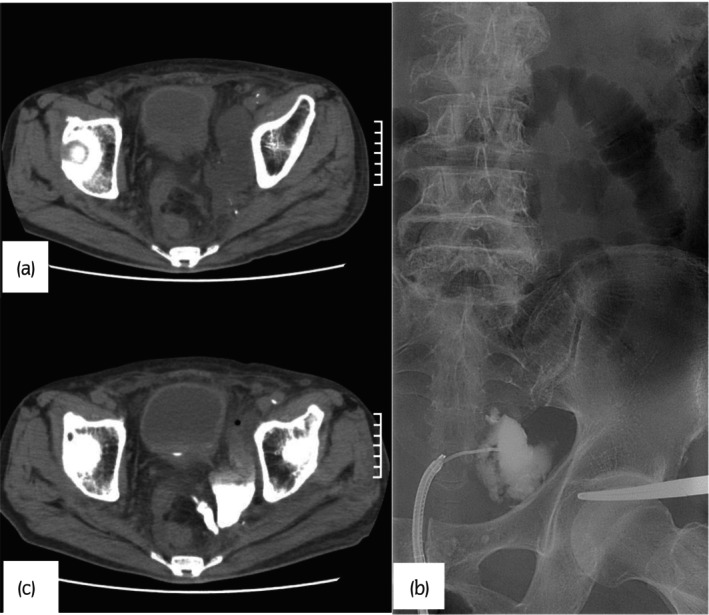
(a) Low‐density polycystic area in the left pelvic cavity on the dorsal side of the sigmoid colon (b) leakage of contrast medium from the left lower ureter to the outside (c) an influx of contrast medium in the low‐density area.

An 80‐year‐old man with stage IIIc rectal cancer (cT3N3M0) underwent robot‐assisted low anterior resection of the rectum. He had lateral lymph node dissection after colostomy and preoperative chemoradiotherapy (5‐fluorouracil and 45 Gy of radiation). Because of the small amount of drainage, the abdominal drain was removed on postoperative day 5. He had a fever on postoperative day 14. A NCCT scan revealed a low‐density polycystic area on the dorsal side of the sigmoid colon in the left pelvic cavity and mild left hydronephrosis (Fig. 1a). The patient was referred to our department for further examination and treatment. His WBC, CRP, and creatinine levels were elevated to 11 700/μL, 26.85 and 2.44 mg/dL, respectively, and urinalysis revealed only mild occult blood. RP showed contrast medium leakage from the left lower ureter (Fig. 1b), preventing ureteral stent placement. After the RP, an NCCT scan revealed an influx of contrast medium in the low‐density area (Fig. 1c). Based on the above examination, the delayed ureteral injury was diagnosed with infected urinoma. After reviewing the surgery video, we observed no apparent indication of ureter rupture or ligation. However, using energy devices near the ureter probably caused ureteral wall necrosis due to thermal injury. Because long‐term nephrostomy management during conservative treatment seemed difficult for the patient, we planned one‐stage laparoscopic drainage of the abscess and ureterocystoneostomy due to the difficulties of puncture drainage for infected urinoma due to the pelvis and sigmoid colon and the risk of increased adhesion and fibrosis surrounding the ureter in the future. As an alternative, we considered open surgery or nephrostomy placement when adhesions were severe.

**Fig. 2:**
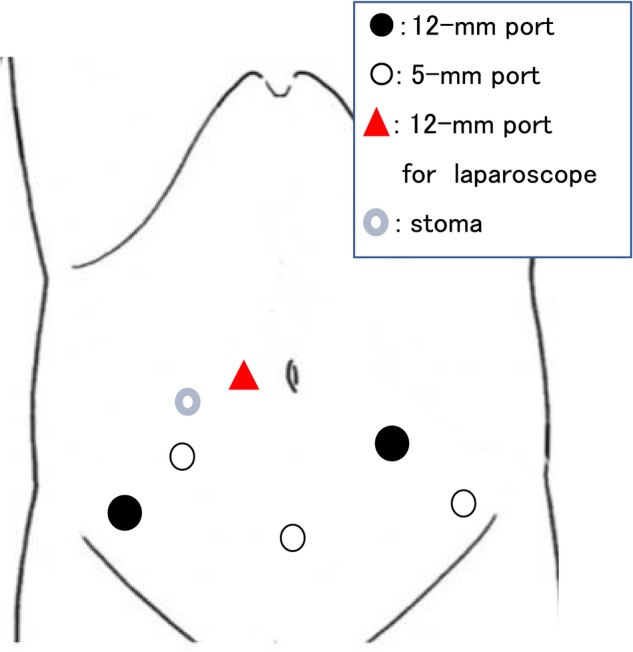
Ports placement during surgery.

**Fig. 3:**
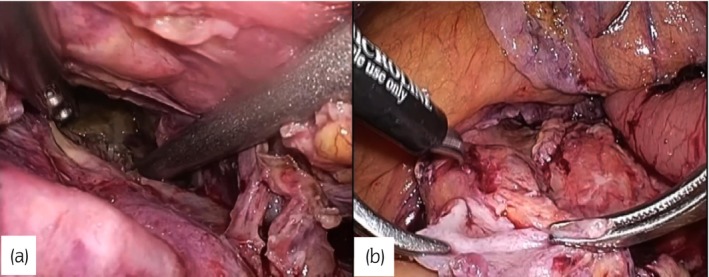
(a) Pus‐filled space was confirmed and opened into the peritoneal cavity (b) a small amount of bleeding from proximal end of ureter incised.

The 12‐mm laparoscope port and other ports were positioned as shown in Figure 2. Some ports used port wounds from previous surgery. Marked adhesion was evident on the outside of the ureter, and a sharp dissection was undertaken. The pus‐filled area was confirmed and opened into the peritoneal cavity after the left ureter was identified inside the sigmoid colon and dissected distally via the dorsal side of the sigmoid colon (Fig. 3a). The bladder was dissected from the anterior abdominal wall, and a 5‐mm suturing port was inserted in the lower abdomen. There was minor bleeding when the ureter was incised, most likely due to radiation or surgery; thus, considering the area with little bleeding as the thermal injury area, the ureter was incised about 5 cm, and a small amount of bleeding was confirmed (Fig. 3b). Following the Psoas‐Hitch method, the peritoneum in front of the promontory and the bladder dome were fixed. A 6‐Fr polyolefin ureteral stent of various lengths was inserted using the port in the lower abdomen through the ureter and bladder. The assistant grasped and pulled the sutured distal end from the port in the lower abdomen, and the bladder and ureter were fixed using the extravesical Lich–Gregoire method. A closed suction drain was installed, and all port sites were closed.

CRP and creatinine levels improved a few days after the operation. On postoperative day 12, cystography was performed, and no apparent leak was detected. The closed drain was removed on postoperative day 19. On postoperative day 26, the urinary catheter was removed, but the patient had a fever on postoperative day 30; hence, the urinary catheter was reinstalled. On postoperative day 42, the urinary catheter and ureteral stent were removed, and he was discharged on postoperative day 52. There has been no increase in the creatinine level or hydronephrosis.

## Discussion

Iatrogenic ureteral injuries are often treated by urologists after abdominal surgery, such as obstetrics and gynecology, digestive surgery, and vascular surgery. The most common cause of iatrogenic ureteral injury is gynecologic surgery (55%–65%), followed by digestive surgery (15%–25%) and urological surgery (11%–25%).^1^, ^2^ Some previous studies have shown that colorectal surgery accounts for 1%–10% of iatrogenic ureteral injuries, but the frequency has recently decreased to <1%.^3^, ^4^, ^5^

According to the EAU guidelines, for postoperative ureteral injuries, ureteral stenting or nephrostomy should be implanted first, followed by ureteral reconstruction if conservative treatment is ineffective.^6^ According to studies, most iatrogenic ureteral injuries improved spontaneously after ureteral stenting or nephrostomy.^7^, ^8^ However, these reports did not include a history of radiation therapy or the cause of injury. The history of radiation therapy has been reported to make conservative cure challenging.^9^ Conservative treatment was challenging in iatrogenic ureteral injury caused by necrosis of the ureteral wall produced by thermal energy.^10^ Although it has been reported that radiation and chemotherapy are associated with a risk of failure in repair of urinary tract injuries,^11^ when there is a history of radiotherapy and possible thermal injury, as in this case, conservative treatment may be more challenging to recover than surgical repairs.

Some reports suggest that surgical repairs for iatrogenic ureteral injuries should be performed 6–12 weeks later. This is because a ureteral injury can develop sequentially if blood flow is impaired, and the passage of time leads to improved edema in the ureteral wall and ligature dissolution.^12^, ^13^ Conversely, some reports recommend early treatment due to the positive result and prevention of decline in QOL with a nephrostomy tube.^14^ There have also been reports of one‐stage laparoscopic drainage and ureteral reconstruction for ureteral injuries associated with large urinoma,^15^ but no reports of ureteral injuries associated with infected urinoma. It is difficult to exclude the possibility that infection‐related inflammation of the ureter and adjacent tissues may affect ureteral reconstruction. In this case, one‐stage drainage and ureteral ureterocystoneostomy of iatrogenic ureteral injury caused by infected urinoma may prevent the decline in QOL associated with nephrostomy tube management, improve the overall condition, and provide early therapeutic intervention for ureteral injury. In this case, the patient continued to stay until the ureteral stent was removed, but in general, if the urethral catheter is removed after surgery and there is no problem urinating, or if the urethral catheter is properly managed, early discharge is possible. However, the extent of the ureteral injury and insufficient blood flow may necessitate nephrostomy tube placement and other ureteral reconstructions, such as ileal ureteric replacement or renal autotransplantation, and there is a risk of ureteral stricture even after repair; therefore, this must be discussed and explained to patients preoperatively.

## Conclusion

In conclusion, this study reported a case of iatrogenic ureteral injury with infected urinoma treated with one‐stage laparoscopic drainage and ureterocystoneostomy.

## Author contributions

Toshifumi Takahashi: Conceptualization; data curation; investigation; writing – original draft; writing – review and editing. Kouhei Maruno: Data curation. Tatsuya Hazama: Data curation. Hideto Ota: Data curation. Yuya Yamada: Data curation. Masakazu Nakashima: Supervision. Kazuro Kikkawa: Data curation. Masahiro Tamaki: Supervision. Noriyuki Ito: Supervision.

## Conflict of interest

The authors declare no conflict of interest.

## Approval of the research protocol by an Institutional Reviewer Board

Not applicable.

## Informed consent

Written informed consent to participate in this study and for the publication of this report was obtained from the patient for ethics approval.

## Registry and the Registration No. of the study/trial

Not applicable.
